# Traumatic tracheobronchial injuries: incidence and outcome of 136.389 patients derived from the DGU traumaregister

**DOI:** 10.1038/s41598-020-77613-x

**Published:** 2020-11-25

**Authors:** David Schibilsky, Arne Driessen, William James White, Rolf Lefering, Thomas Paffrath, Bertil Bouillon, Tobias Walker, Christian Schlensak, Manuel Mutschler

**Affiliations:** 1 University Heart Center Freiburg, Bad Krozingen, Hugstetterstr. 55, 79106 Freiburg, Germany; 2 Faculty of Medicine, University Freiburg, Freiburg, Germany; 3 Klinik für Orthopädie, Universitätsklinik RWTH Aachen, Pauwelsstrasse 30, 52074 Aachen, Germany; 4 Department of Trauma and Orthopaedic Surgery, Chelsea and Westminster Hospital, NHS Foundation Trust, 369 Fulham Road, London, SW10 9NH UK; 5 IFOM-Institut für Forschung in der Operativen Medizin, Universität Witten/Herdecke, Ostmerheimerstrasse 200, 51109 Köln, Germany; 6 Klinik für Orthopädie, Unfallchirurgie & Sporttraumatologie, Krankenhaus Köln Merheim, Universität Witten/Herdecke, Ostmerheimerstrasse 200, 51109 Köln, Germany; 7 Universitätsklinik für Thorax-, Herz- und Gefäßchirurgie, Hoppe-Seyler-Straße 3, 72076 Tübingen, Germany

**Keywords:** Trauma, Orthopaedics, Outcomes research, Respiratory signs and symptoms

## Abstract

To describe the incidence, therapy and outcome of traumatic tracheobronchial injuries (TTBI) in trauma patients with multiple injuries derived from the DGU TraumaRegister. We analyzed the data on all patients listed on the TraumaRegister DGU (TR-DGU) in Germany between 2002 and 2015 aged 16 years or older and with an Injury Severity Score (ISS) of ≥ 9. We analyzed the data on 136,389 trauma patients, 561 of whom had suffered tracheobronchial injuries (0.4%). The majority were male (73.4%) and had a mean age of 43.7 years. In total, 84.0% of all TTBI injuries occurred secondary to blunt trauma, caused mainly by accidents (71.2%). TTBI was accompanied by several concomitant thoracic injuries such as pneumo- (41.2%) and hemothorax (23.2%), lacerations (7.8%) and contusions (32.3%) of the lung, as well as multiple rib fractures (29.6%). The severity of injury was classified via the abbreviated injury scale (AIS): 39.3% with AIS = 3, 51.3% with AIS = 4 and 60% with AIS = 5 patients underwent surgical interventions. The mortality of patients with tracheobronchial injuries was higher: 24.6%, versus 13.7% in all patients (control group). This high percentage reflects their generally severe injury burden through concomitant injuries. The incidence of TTBI in this large cohort of trauma patients is very low. However, its high mortality rate emphasizes its importance. Mortality was associated with higher ISS and AIS scores. Higher rates of concomitant injuries were therefore associated with a higher mortality rate. TTBI injuries revealed a higher rate of progression to surgical management, with 35% undergoing surgery within the first 24 h. This excessive mortality rate demonstrates a high overall injury burden in patients with TTBI and high mortality of associated injuries. A surgical intervention’s impact on mortality cannot be assessed in this study, as it would need to be investigated in a case-matched study.

## Introduction

Acute traumatic tracheobronchial injuries (TTBI) resulting from blunt or penetrating trauma are rare but severe life-threatening traumatic injuries. Their incidence and mortality have not been well described to date. Most of the underlying evidence has relied on single-center experiences or case reports. The potentially high rate of prehospital mortality may also be a factor behind this this lack of published data^1,2^.

The cause of trauma varies widely among published series. Several case-series reporting mainly blunt or penetrating trauma^3–5^ have been published. Mortality rates of patients with TTBI reporting from specialized trauma centers^6,7^ range from 10 to 20%. The outcome of patients suffering severe blunt chest trauma was worsened by suffering additional (more than 2) extrathoracic injuries. However, as the aforementioned studies were published over two decades ago, widely-used trauma scores were not employed in their assessments and reports, thus limiting their comparability^8^.

There is still controversy as to whether tracheobronchial injuries require surgical management. Reports on the non-surgical management of single, small-to-moderate sized lesions (< 2 cm) with favorable outcome have appeared^9,10^. The operative management of lesions > 2 cm tends to favor more positive outcomes; mortality is mainly associated with concomitant trauma injuries^3,6^. Complex injuries might require complex surgical reconstruction such as bronchial end-to-end anastomosis or pneumonectomy^11^. Complete recoveries have, however, been reported in patients with lesions > 2 cm without any surgical intervention^12^.

Therefore, the correct diagnosis of TTBI can have a serious implication. Chest CT can be utilized to diagnose and further characterize the site and size of TTBI in most patients after posttraumatic or iatrogenic injury^13^. Bronchoscopy has been applied to confirm tracheobronchial injury patterns and facilitate surgical interventions^3,5^ in most published series.

All the published data and current therapeutic recommendations are based on the experience of single centers reporting on very few patients in their respective series.

This study aims to assess the incidence, trauma mechanism, trauma severity, therapeutic strategies and outcome of traumatic TTBI within the last decade.

## Methods

The DGU TraumaRegister of the German Trauma Society (Deutsche Gesellschaft für Unfallchirurgie, DGU) was founded in 1993. The aim of this multicenter database is the pseudonymized and standardized documentation of severely injured patients.

Data was collected prospectively at four consecutive time stages from the site of the accident until discharge from hospital, namely: (A) pre-hospital phase, (B) emergency room and initial surgery, (C) intensive care unit and (D) discharge. Documentation includes detailed information of patient demographics, injury pattern, comorbidities, pre- and in-hospital management, course on intensive care unit, relevant laboratory findings including transfusion data and each patient’s outcome. The inclusion criteria were admission to hospital via the emergency room with subsequent ICU/ICM care or reaching the hospital with vital signs and before admission to ICU.

The participating hospitals are primarily located in Germany (90%), but a rising number of hospitals in several different countries contribute data to this database (currently Austria, Belgium, China, Finland, Luxembourg, Slovenia, Switzerland, the Netherlands, and United Arab Emirates). Approximately 25,000 cases from more than 600 hospitals are entered in the database per year.

Participation in the DGU TraumaRegister is voluntary and open to all trauma hospitals. The dataset is freely accessible for clinicians and clinics wishing to analyze its data.

For hospitals signed up to the TraumaNetzwerk DGU collaboration, entry of at least one basic data set is obligatory for reasons of quality assurance.

The present study is in line with the publication guidelines of the DGU TraumaRegister and is registered as TR-DGU project ID 2013-008.

All methods concur with relevant guidelines and regulations. More information is available online under http://www.traumaregister-dgu.de.

### Data analysis

Datasets on all multiply-injured trauma patients treated in a trauma center between 2002 and 2015 were analyzed. Inclusion criteria were age ≥ 16 years, primary admission to one of the affiliated hospitals, as well as an Injury Severity Score (ISS) ≥ 9 points.

The tracheobronchial injuries addressed in this study included tracheal, main bronchial, and minor bronchial injuries. Individual patients were identified via the specific abbreviated injury scale (AIS) code (AIS version 2005) related to the injuries—AIS codes 3402xx and 3404xx for the upper airway; AIS 3416xx, 4401xx, 4402xx, and 4426xx for the lower airway. We analyzed patient demographics, injury characteristic, injury pattern, trauma mechanism, therapeutic strategy and specific outcome. To detect potential differences, TTBI patients were compared to a cohort of trauma patients from Germany (ISS ≥ 9, age ≥ 16 years, primary admission to trauma centre between 2002 and 2015) recorded in the DGU TraumaRegister (control group).

### Definition of multiple organ failure (MOF)

Organ function was assessed by using the “Sequential Organ Failure Assessment” (SOFA) score^1,2^. This score was designed to simply assess the incidence and severity of organ dysfunction in critically ill patients. Three or four SOFA points were considered as organ failure. For each organ or function, i.e., lung, liver, kidney, coagulation system, cardiovascular system, and central nervous system, the number of days of documented organ failure during the hospital stay was recorded. In addition, the number of days suffering multiple organ failure (MOF; at least two failing organs) was recorded.

### Statistical methods

Data is presented as mean ± standard deviation (SD) for continuous variables or percentages (%) for categorical variables. In case of skewed distributions, the median is presented in addition. Due to the control group’s large sample size, statistical uncertainty was only relevant in the target population with tracheobronchial injuries. We relied on Fisher’s Exact test for categories, and the Mann–Whitney U-test for continuous measurements.

All data were analyzed using IBM SPSS (IBM SPSS 23, Chicago, USA).

## Results

A total of 136,389 patient records derived from 640 hospitals were identified for the present analysis following the aforementioned inclusion and exclusion criteria. The overall incidence rate for traumatic tracheobronchial injuries was 0.4% (561 of 136,389).

General demographics and detailed information on overall injury severities are illustrated in Tables 1 and 2. Patients were predominantly male (73.4%) and aged a mean 43.7 years. In total, 84% of all TTBI patients suffered a blunt trauma, and the underlying cause was predominantly accidents in 71.2%, with 22.2% caused by suicide attempts (22.2%). Car accidents (26.8%), falls (23.7%) and motorcycle accidents (17.7%) were the leading causes of accidents in this group (Table 1).

**Table 1:** Demographics and trauma mechanisms of all patients, and of all suffering tracheobronchial trauma.

	Tracheobronchial injury	Control
Total number	561	135.828
**Demographics**		
Male gender [percentage of total (n)]	73.4 (n = 409)	71.1 (n = 96.290)
Age (years)	43.7 (SD = 19.5)	50.6 (SD = 21.0)
**Mechanism of Trauma [percentage of total (n)]**		
Penetrating	16% (n = 87)	4.4% (n = 5.433)
**Cause of injury [percentage of total (n)]**		
Accident	71.2% (n = 409)	92.7% (n = 125.912)
Violence	6.6% (n = 36)	2.3% (n = 3.124)
Suicide	22.2% (n = 121)	4.8% (n = 6.520)
**Cause of accident [percentage of total (n)]**		
Car accident	26.8% (n = 136)	24.8% (n = 33.685)
Motorcycle accident	17.7% (n = 86)	14.5% (n = 19.695)
Bicycle accident	6.2% (n = 30)	8.7% (n = 11.817)
Pedestrian accident	3.1% (n = 15)	6.9% (n = 9.372)
Fall < 3 m	17.5% (n = 85)	17.3% (n = 23.498)
Fall > 3 m	6.2% (n = 30)	20.1% (n = 27.301)

**Table 2:** Injury severity and concomitant injuries (ISS: Injury Severity Score; NISS: New Injury Severity Score; AIS: Abbreviated Injury scale) of all patients and of all those with tracheobronchial trauma.

	Tracheobronchial injury	Control
**Trauma scores**		
ISS	30.7 (16.2)	21.5 (12.3)
NISS	38.0 (18.7)	26.9 (14.8)
**Relevant injuries (abbreviated injury scale (AIS) > 3 [percentage of total (n)]**		
AIS head	65.4% (n = 367)	41.1% (n = 55,789)
AIS thorax	70.8% (n = 397)	46.3% (n = 62,888)
AIS abdomen	14.8% (n = 83)	12.7% (n = 17,250)
AIS extremities	24.2% (n = 136)	31.2% (n = 42,378)
**Specific other injuries or sequelae [percentage of total (n)]**		
Diaphragm	2.1% (n = 12)	0.8% (n = 1.086)
Esophagus	1.2% (n = 7)	< 0.1% (n = 58)
Lung laceration	7.8% (n = 44)	3.0% (n = 4.075)
Lung contusion	32.3% (n = 181)	26.0% (n = 35.315)
Multiple rib fractures	29.6% (n = 166)	26.9% (n = 36.538)
Hematothorax	23.2% (n = 130)	11.6% (n = 15.756)
Pneumothorax	41.2% (n = 231)	20.2% (n = 27.437)
**Airway injuries [percentage of total (n)]**		
Upper airway	29.4% (n = 165)	
Lower airway	72.0% (n = 404)	
Both	1.4% (n = 8)	

### Injury severity and concomitant injuries

Patients with TTBI revealed higher overall injury severities than other trauma patients listed in the DGU TraumaRegister. This was reflected by their higher injury severity scores (ISS) and new injury severity scores (NISS) values. Head, thorax and abdomen injuries were more frequent in these patients than in the total trauma population (Table 2).

TTBIs were accompanied by several concomitant thoracic injuries and sequelae, as depicted in Table 2. We noted that it was mainly the sequelae of thoracic injuries such as pneumo- and hemothorax (64.4%), followed by thoracic injuries like lacerations or lung contusions (40.1%), serial rib fractures and flail chests (29.6%) that also revealed an association. Structures adjacent to the tracheobronchial system such as the diaphragm (2.1%) and esophagus (1.2%) were only injured in a lowl percentage. Injury to the lower airway exclusively occurred in 72% of patients (Table 2).

### Pre-hospital and ER management

The pre-hospital management of TTBI patients is illustrated in Table 3. Approximately half of TTBI patients (47.6%) received a chest tube. A chest tube was put in place in a pre-hospital setting in 14.6% of the patients, and in 33% during the initial emergency room management.

**Table 3:** Non-surgical treatment of patients with tracheobronchial injuries and among the control group (CPR: cardiopulmonary resuscitation).

	Tracheobronchial injury	Control
**Prehospital treatment [percentage of total (n)]**		
Intubation	58.9% (325)	33.1%
**No intubation**		
Chest tube	14.6% (n = 57)	4.2% (n = 5.705)
Volume amount (ml)	1147 (SD = 877)	860 (SD = 739)
Catecholamines	19.2% (n = 75)	8.6% (n = 11.681)
CPR	10.7% (n = 59)	3.3% (n = 4.482)
Helicopter transport	28.4% (n = 154)	25.7% (n = 34.908)
Time from trauma to admission (min)	66 (SD = 29)	64 (SD = 29)
**Emergency department treatment [percentage of total (n)]**		
Intubation	33% (n = 126)	
**No intubation**		
Chest tube	33% (n = 126)	14.7% (n = 19.967)
Blood transfusion	27.7% (n = 153)	13.2% (n = 17.929)
Whole body CT scan	72.3% (n = 401)	72.4% (n = 98.339)

Pre-hospital intubation (58.9% vs. 33.1%), cardiopulmonary resuscitation (10.7% vs. 3.3%), the average amount of volume administered during initial resuscitation (1147 ml vs. 860 ml), and vasopressor use (19.2% vs. 8.6%) were significantly higher in TTBI patients. The number of TTBI patients requiring blood transfusions during initial ER management was over twice that of the control group (27.7% vs. 13.2%).

### Surgical treatment and outcome

More severe injuries were linked to a greater need for surgical intervention among TTBI patients. 39.3% of all patients with an AIS thorax = 3, 51.3% of all patients with an AIS thorax = 4 and 60% of all patients with an AIS thorax = 5 underwent surgical interventions to alleviate their TTBI.

Considering that our TTBI group includes fatal injuries (effectively excluding those individuals from the operative management group), the mortality rate in patients without surgery was 25% (n = 58 out of 231). The mortality rate of patients who underwent surgery within 24 h was 20% (n = 28 out of 140), and 4% of patients operated on later than 24 h (n = 1 out of 26).

As we had to consider an incomplete data set (no standard documentation available) in 164 patients without documented surgery, the mortality rate was 30.5% (50 out of 164 patients) in the TTBI patients.

An isolated TTBI (TTBI and AIS head/abdomen/extremities score ≤ 2) was present in 86 of 561 patients (their mortality rate was very low [2.3% (n = 2) (95% CI 0.2–8.4%)], whereas the mortality rate of those TTBI patients with concomitant injuries was 28.6% (n = 136).

Prehospital intubation was performed in 58.9% (standard documentation only): their mortality rate was 36.6%. The mortality rate without intubation was 8.4%.

The emergency departments’ (ED) intubation rate was 35.6% (n = 136) and their mortality rate 24.3%.

The mortality rate of patients who did not experience prehospital or ED chest tube placement was 5.4%. The mortality rate rises sharply in those patients undergoing prehospital chest tube application (10.4%), and is even steeper (up to 31.4%) in those patients who had a chest tube put in place in the ED (Table 3).

Table 4 A/B provides detailed information on surgery time-points. AIS 5 patients underwent the highest proportion of operations (60%), whereas 51% of AIS 4 patients and 39% of AIS 3 patients underwent surgery.

**Table 4:** A: Surgical treatment of patients with tracheobronchial injuries, B: Surgical treatment and mortality according to the Abbreviated Injury Scale (available in the standard documentation only).

(A)	Surgery of tracheobronchial injury		
	Airway injury	Upper airway injury	Lower airway injury
No. of cases	397	128	274
Surgery < 24 h	35% (n = 140)	32% (n = 41)	37% (n = 102)
Surgery > 24 h	7% (n = 26)	9% (n = 12)	5% (n = 14)
No surgery	58% (n = 231)	59% (n = 75)	58% (n = 158)

(B) Surgery according to abbreviated injury scale (AIS) [percentage of total (n)]			Mortality (percentage of total)
AIS 2	31.2% (29 of 93)		19.5%
AIS 3	39.3% (64 of 163)		19.2%
AIS 4	51.3% (39 of 76)		21.4%
AIS 5	60% (33 of 55)		51.2%

*AIS* Abbreviated Injury Scale.

During their clinical course, 35.5% (117 of 397) of TTBI patients developed multiple organ failure (MOF) and 34.2% (113 of 397) acute lung failure (standard documentation only—Table 5). Overall hospital mortality rate in patients with TTBI was 24.6%: 17.1% of those died within the first 24 h after admission, thereby revealing a mortality rate more than twice that of non-TTBI patients (7.0%).

**Table 5:** Outcome of all patients and of all patients with tracheobronchial trauma (RISC II: Revised Injury Severity Classification Score).

	Tracheobronchial injury	Control
**Overall outcome**		
Mortality	24.6% (n = 138) (RISC II 25.1%)	13.7% (RISC II 13.4%)
24 h Mortality	17.1% (n = 96)	7.0% (n = 9.508)
Multiorgan failure	35.5% (n = 117)	24.7% (n = 33.550)
Lung failure	34.2% (n = 113)	18.8% (n = 25.536)
**Hospital stay in survivors**		
Length of stay (LOS) (days)*	25.4/18 (SD = 26.8)	21.4/16 (SD = 20.6)
ICU stay (days)*	14.3/10 (SD = 14.4)	8.8/4 (SD = 11.9)
Ventilator time (days)*	11.4/7 (SD = 12.1)	9.1/4 (SD = 11.5)
**Mortality TTBI with or without concomitant injuries**		
Isolated TTBI (AIS head/abdomen/extremities ≤ 2)	2.3% (n = 2/86)	
TTBI and AIS (head/abdomen/extremities) ≥ 3	28.6% (n = 136/475)	

Of the 561 TTBI patients, 138 (24.6%) died. Those patients were older (47 vs. 43 years), had a a higher ISS (45 vs. 26) and more frequent serious (AIS > 3) head (73% vs 63%) and thoracic (88% vs. 75%) traumas (Table 6).

**Table 6:** Characterization of survivors vs. non-survivors among patients with tracheobronchial injuries.

561 patients with TBI	Survivor	Non-survivor	P value
No. of patients	423 (75.4%)	138 (24.6%)	
Age (years)	43 (SD = 19)	47 (SD = 21)	0.113
Sex (males)	318 (76%)	91 (73%)	0.57
Injury Severity Score (ISS)	26 (SD = 13)	45 (SD = 17)	< 0.001
Serious head injury (AIS > 3)	267 (63%)	100 (73%)	0.050
Serious thoracic trauma (AIS > 3)	275 (75%)	122 (88%)	< 0.001
Prehospital intubation	206 (50%)	119 (86%)	< 0.001
Blood transfusion (emergency room)	88 (21%)	65 (50%)	< 0.001

Length of stay (LOS) was longer: 25.4 days for TTBI patients (survivors only) compared to 21.4 days in the control group. The number of days on mechanical ventilation (11.4 vs. 9.1 days) and LOS on the ICU (14.3 vs. 8.8 days) were also higher for TTBI patients (survivors only) than the control group.

## Discussion

This study describes the incidence, clinical course, and outcome of patients who suffered a TTBI and are documented in the DGU TraumaRegister. With over 160,000 patients analyzed, this is one of the largest trauma registries in the world. This analysis is the first description of the incidence and characteristics of TTBI patients among a large cohort of trauma patients.

The incidence of TTBI in this large cohort of trauma patients is low (0.4%); it affected predominantly young male patients suffering from blunt trauma (84%) caused mainly by accidents (71.2%). The mortality of patients undergoing surgery within the first 24 h after the impact is 20%. If surgery is delayed (> 24 h), mortality is 4%. We observed the highest mortality (25%) in the group without any surgical intervention, which reveals the heavy injury burden caused by concomitant injuries.

It is possible that these findings also reflect the urgent treatment of potentially lethal injuries other than TTBI. TTBI treatment might have been postponed due to the greater initial urgency of treating more severe concomitant injuries.

Patients with isolated TTBI demonstrate relatively low mortality compared to overall mortality. It is not the fatality of TTBI alone that leads to worse outcomes, but rather the overall heavy injury burden.

Furthermore, we noted that mortality was associated with a higher AIS score, emphasizing the need for potentially life-saving surgical procedures within the first 24 h in 35% of these patients.

In-hospital mortality in published series ranges from 9%^9^, 19%^3^ to 20%^7^ in recent study cohorts.

Our study’s higher mortality might be multifactorial. The TTBI group’s mortality rate within the first 24 h post-injury was higher than the control group’s, a factor that reveals the significant mortality risk associated with tracheobronchial injuries.

Only one study reported injury severity scores (ISS) of 24.3 ± 8.6 for survivors and 34.7 ± 8.8 for non-survivors^5^.

The TTBI patients’ days on ventilator (11.4 vs. 9.1 days) and LOS on the ICU (14.3 vs. 8.8 days) were different than the control group’s—perhaps attributable to complex injury patterns requiring extended artificial pulmonary ventilation.

In contrast, our analysis reveals a mean ISS of already 30.7 points (survivors 25.9 points; non-survivors 45.4 points) in all patients with TTBI. Although their injury severity is higher through their concomitant injuries, we are unable to differentiate between TTBI and any other cause of death^5^.

Furthermore, the nationwide inclusion of trauma patients from level-1 to level-3 trauma centers in the DGU TraumaRegister means that patients were included who were treated in either specialized centers or non-cardiothoracic surgery units. This study therefore reflects a real-world scenario rather than the experience of highly specialized centers. Furthermore, the good outcomes of patients in published case series are probably affected by reporting bias.

The trauma causes differ among these studies. Whereas a cohort of Turkish patients suffered TTBI mainly from blunt trauma^5^, a large cohort from Texas, USA suffered mainly penetrating TTBI^3^—a factor likely related to the higher incidence of gunshot wounds in the US series. The blunt trauma mechanisms in most of our cohort match those experienced in other European centers.

Common CT scan algorithms that are not technically demanding can help diagnose TTBI with great certainty^14^. Thus, in case of clinical suspicion, standard diagnostics should be applied to identify or rule out TTBI.

This study shows that patients with higher AIS scores also reveal a higher incidence of surgically managed TTBI (Table 4). Despite this surgical intervention, mortality remains high, especially in the group of patients needing surgery within 24 h. Concomitant injuries most likely contributed to the TTBI patients’ high mortality—also reflected by the higher ISS and more frequent severe (AIS > 3) head injuries among the non-survivors in the TTBI group.

Due to this study’s database nature, we can delineate the incidence and general mortality of different patient subgroups. We were able to rely on widely used trauma severity scores to match the severity of concomitant injuries and TTBI with these patients’ outcomes.

As we have been unable to provide detailed information on the specific nature of TTBI, diagnostics, or particular treatments in this study, we are not in a position to comment on the value of certain therapeutic regimes in given clinical situations.

Still, the diagnosis of TTBI should not be underrated. For patients presenting a clinically potential TTBI, standard diagnostics should be carried out to rule out such injuries. The diagnostic tools for assessing TTBI such as standard CT scan or bronchoscopy are widely available and implemented in many standard ER algorithms. Full awareness of TTBI’s life-threatening consequences should mean earlier referral to specialized centers. The mortality rates such centers report are quite positive. It remains unclear whether there is a survival benefit for patients treated at specialized centers compared to general trauma hospitals. The published evidence, including case series, lacks accurate descriptions and documentation. However, it is debatable whether specialized centers enable better outcomes than general district hospitals^9,14^. As the high mortality of TTBI patients is associated with their heavy multiple-injury burden, direct referral to a specialized center is perhaps advisable for them: note that non-survivors revealed a higher ISS and more frequent severe (AIS > 3) head and thoracic traumas. Furthermore, our data show that TTBI patients suffer more often from esophageal and diaphragmatic injuries. These (often occult) pathologies should therefore be ruled out in all patients with suspected TTBI.

## Conclusion

Patients with TTBI demonstrate a high associated mortality rate that appears to be attributable to their heavy overall injury burden and the severity of associated injuries.

Early surgery in patients with TTBI tends to reflect their heavy injury burden and severity of associated injuries. Although TTBI’s incidence is low, standardized diagnostic algorithms and assessment tools should be employed when admitting trauma patients, focusing on occult concomitant injuries such as esophageal and diaphragmatic injuries, which were more frequent among TTBI patients than in our the control group.

What the most promising treatment strategy for handling concomitant injuries and TTBI in severely injured trauma patients is remains unclear.

Still, questions regarding TTBI management remain. The indications for surgery itself have triggered controversy. However, this analysis cannot clarify this issue due to missing data in the registry on the severity and location of TTBI, surgical specifics, and management techniques.

Multicenter investigations applying structured diagnostic and treatment algorithms would help us clarify issues such as the indications for, timing, and appropriate methods of surgery. We hypothesize that the early treatment of trauma patients presenting concomitant injuries and tracheobronchial injuries in trauma centers would have a generally positive effect on such patients’ outcomes.

## References

[1] Baker S, O’Neill B, Haddon W, Long W (1974). The Injury Severity Score: A method for describing patients with multiple injuries and evaluating emergency care. J. Trauma.

[2] Burke JF (1962). Early diagnosis of traumatic rupture of the bronchus. JAMA.

[3] Rossbach MM (1998). Management of major tracheobronchial injuries: A 28-year experience. Ann. Thorac. Surg..

[4] Greenspan L, McLellan B, Greig H (1985). Abbreviated Injury Scale and Injury Severity Score: A scoring chart. J. Trauma.

[5] Balci AE, Eren N, Eren S, Ulkü R (2002). Surgical treatment of post-traumatic tracheobronchial injuries: 14-year experience. Eur. J. Cardiothorac. Surg..

[6] Gabor S (2001). Indications for surgery in tracheobronchial ruptures. Eur. J. Cardiothorac. Surg..

[7] Barmada H, Gibbons JR (1994). Tracheobronchial injury in blunt and penetrating chest trauma. Chest.

[8] Svennevig JL (1986). Prognostic factors in blunt chest trauma. Analysis of 652 cases. Ann. Chir. Gynaecol..

[9] Kiser AC, O’Brien SM, Detterbeck FC (2001). Blunt tracheobronchial injuries: Treatment and outcomes. Ann. Thorac. Surg..

[10] Kuhne CA (2005). Nonoperative management of tracheobronchial injuries in severely injured patients. Surg. Today.

[11] Zhao Y (2007). Effective management of main bronchial rupture in patients with chest trauma. Thorac. Cardiovasc. Surg..

[12] Gómez-Caro A (2006). Role of conservative medical management of tracheobronchial injuries. J. Trauma.

[13] Scaglione M (2006). Acute tracheobronchial injuries: Impact of imaging on diagnosis and management implications. Eur. J. Radiol..

[14] Le Guen M (2007). Chest computed tomography with multiplanar reformatted images for diagnosing traumatic bronchial rupture: a case report. Crit. Care.

